# The Root Membrane Technique: Human Histologic Evidence after Five Years of Function

**DOI:** 10.1155/2017/7269467

**Published:** 2017-11-22

**Authors:** Miltiadis E. Mitsias, Konstantinos D. Siormpas, Georgios A. Kotsakis, Scott D. Ganz, Carlo Mangano, Giovanna Iezzi

**Affiliations:** ^1^Department of Periodontology and Implant Dentistry, College of Dentistry, New York University, New York, NY 10010, USA; ^2^Private Practice, Athens, Greece; ^3^Private Practice, Larissa, Greece; ^4^Division of Periodontology, School of Dentistry, University of Minnesota, Minneapolis, MN 55455, USA; ^5^Rutgers School of Dental Medicine, Newark, NJ 07103, USA; ^6^Hackensack University Medical Center, Hackensack, NJ 07601, USA; ^7^Private Practice, Fort Lee, NJ 07024, USA; ^8^Department of Dental Sciences, Vita and Salute University S. Raffaele, 20132 Milan, Italy; ^9^Department of Oral and Biotechnological Science, University G. D'Annunzio, 66013 Chieti, Italy

## Abstract

**Background:**

The “root membrane” (RM) is a technique that has become popular among implantologists for placement of immediate implants in the anterior maxilla.

**Purpose:**

To present histologic evidence of an immediate implant placed in the human anterior maxilla, according to the RM technique, and retrieved after five years.

**Methods:**

A fixture, along with the surrounding tissues, was retrieved from the anterior maxilla of a 68-year-old patient, who had been treated five years earlier with immediate implant placement and RM technique. The specimen was processed for histologic/histomorphometric evaluation.

**Results:**

The buccal bone plate was maintained without any resorption; a healthy periodontal ligament was evidenced. The implant showed osseointegration, with a high percentage of bone-to-implant contact (BIC = 76.2%). With regard to the space between the RM and the implant, the apical and medial thirds were filled with compact, mature bone; the coronal third was colonized by noninfiltrated connective tissue.

**Conclusions:**

The RM technique appears to be effective in preventing bone resorption of the buccal bone plate of the human anterior maxilla, five years after the placement of an immediate implant.

## 1. Introduction

To date, the rehabilitation of the anterior maxilla with postextractive single implants represents a successful treatment procedure characterized by high survival rates, as evidenced by several short- [1, 2] and long-term [3, 4] clinical studies.

However, this surgical procedure remains complex for the surgeon because it can be difficult to obtain a prosthetic restoration that mimics the emergency profile and the appearance of the natural, contralateral tooth, in perfect symmetry with it [2–4].

In order to achieve a completely integrated restoration in the aesthetic areas of the anterior maxilla that is indistinguishable from the natural contralateral tooth, it is necessary to preserve and maintain the architecture of the hard and soft tissues [5, 6].

Unfortunately, as has been known for some time, tooth extraction triggers a physiological and unavoidable bone resorption process: in fact, tooth loss leads to a loss of the periodontal ligament and the vascular vessels associated with it [7–9]. Since these vessels help to nourish the buccal bone plate, especially in the anterior maxilla where the delicate and thin buccal bone receives most of its vascular contribution from the periodontal ligament, it is intuitive that this loss inevitably triggers a bone resorption, which is concentrated in the first four to six months after the extraction [7–9]. This resorption of the buccal bone plate, which may be particularly marked in relation to the bone anatomy and biotype of the patient, results in a contraction of the overlying soft tissues and therefore in an aesthetic problem [4, 7, 8, 10, 11]. In fact, it can be difficult or impossible for the clinician to fabricate a restoration that mimics the soft tissue architecture of the natural, contralateral tooth [4, 8, 10, 11].

Over the years, various surgical techniques have been proposed to reduce or compensate for the effects of bone resorption triggered by the tooth extraction and in order to allow the surgeon and prosthodontist to deliver a single-tooth restoration capable of perfect aesthetic integration in the anterior maxilla [8, 12–17]. Among these techniques, there are several variants of alveolar socket preservation [13, 14], guided bone regeneration (GBR) with membranes [15], and/or augmentation procedures with different grafting materials [16], as well as gingival grafts [17].

All these techniques may, through different methods, limit or mask the unpleasant effects of bone resorption of the buccal bone plate (and the related contraction of the overlying soft tissues), allowing for successful aesthetic rehabilitation in the anterior areas [8, 12–17]. However, none can completely eliminate the problem, which is inevitably linked to, and caused by, the extraction of the tooth [13, 18].

A possible alternative to these traditional techniques is offered by the so-called “socket shield” technique, introduced for the first time by Hürzeler and colleagues in 2010 [19]. This technique consists of beheading the crown of the compromised tooth and then sectioning the root mesiodistally; the palatal portion of the root is then extracted, leaving in situ the buccal portion, directly in contact with the buccal bone plate [19]. This portion, defined by the authors as the “socket shield,” is reduced in thickness, concaved, and left 1 mm above the bone crest, taking care to avoid its mobilization [19, 20]. Therefore, it is possible to insert a postextractive implant palatally to the shield, and the gap between the shield and the fixture may (or may not) be filled with particulate graft material [19–21].

The purpose of this technique is to maintain and preserve that portion of periodontal ligament that nourishes the buccal bone plate, thus avoiding the triggering of the physiological bone resorption caused by the tooth extraction [19–21]. The maintenance of the periodontal ligament and hence of the associated blood vessels can, in fact, prevent resorption of the buccal bone and therefore the contraction of the overlying soft tissues [20–22].

The socket shield technique is particularly applicable for the aesthetic areas of the anterior maxilla, in the case of teeth that cannot be restored due to traumas (crown fractures) or destructive caries [22]. Conversely, it cannot be applied to teeth with present (or past) periodontal disease, or with mobility or widening of the periodontal ligament. Moreover, it is not applicable for teeth with vertical root fractures or horizontal fractures below bone level, or to teeth with external/internal resorptions [22].

In the last few years, the socket shield technique has spread rapidly [22], and a number of research groups have published studies proposing surgical variants [20, 21, 23–27]. However, there is still no consensus as to whether or not the space between the shield and the implant (if present) should be grafted. Gluckman et al. [24, 25] who have renamed this technique “partial extraction therapy” (PET) suggest that if present, the gap between the fixture and the shield should always be grafted with particulate graft material. In contrast, Siormpas and Mitsias [26, 27] support the concept that it is not necessary to graft this space; in addition, since the essence of this method consists of preserving the periodontal ligament and hence the associated vascular contribution, they refer to this technique with the name “root membrane” (RM).

Beyond these studies, it is important to note that scientific evidence pertaining to this method is currently scarce since there are only a few clinical studies available in the literature [20, 23, 26, 27]. Most of these are reports on a small number of patients [20, 23, 27], and there are only two retrospective studies with a maximum follow-up period of five years [23, 26]. For this reason, although all the aforementioned clinical studies report positive outcomes, little is known about the possible failures and/or complications associated with this method in the long term [22, 26].

Furthermore, the definitive validation of the effectiveness of this technique must necessarily go through the evaluation of the histologic results obtained with humans in the medium and long term. To date, in fact, there are few histological reports in the literature, and all these are animal studies [19, 20, 28].

Hence, the aim of our present study is to present histologic evidence of an immediate implant placed in the human anterior maxilla, according to the RM technique, and retrieved after five years of function, with the surrounding hard and soft tissues. The analysis of a human histologic sample in which the RM technique has been intentionally used and the shield has remained in situ for a period of five years can improve the understanding of the effectiveness of this new surgical approach in preserving the buccal bone plate and therefore the aesthetic outcomes.

## 2. Methods

### 2.1. Patient Information and Treatment Plan

A 63-year-old male underwent surgery in January 2012 to replace a maxillary lateral incisor (#12) no longer restorable due to a traumatic injury that resulted in a horizontal fracture. The patient was informed that the fracture made the tooth unrestorable and was offered different treatment options, including a traditional bridge on natural teeth (#11–#13) and a single-tooth restoration supported by a dental implant. The patient opted for the latter solution and asked to be treated in a single surgical session, including tooth extraction and immediate implant placement. At the clinical and radiographic examination, the residual root (#12) appeared stable and the bone levels maintained; for this reason, the surgeon (Konstantinos D. Siormpas) decided to perform an immediate implant placement with a root membrane (RM) technique, with the aim of preserving the bone and soft tissue levels and therefore achieving a better aesthetic result. Before the surgery, the patient received a thorough explanation of the treatment procedure and signed a detailed informed consent form.

### 2.2. Immediate Implant Placement with the Root Membrane Technique

The patient was asked to rinse with chlorhexidine 0.2%, 30 minutes before the surgery. Local anaesthesia was administered by infiltration. The crown of the unrestorable tooth (#12) was beheaded with a diamond bur under copious irrigation and then removed. The remaining tooth structure was then levelled 1 mm above the bone tissue crest using the same bur. The root was next sectioned mesiodistally, using a carbide bur, and the palatal portion was separated and gently retrieved using a periotome. The buccal portion of the root was then concaved and thinned, using a carbide bur, in order to follow the profile of the buccal bone. Care was taken during all these procedures not to mobilize the buccal portion of the root and not to damage the socket walls. The goal was to have an implant bed consisting of mesial, distal and palatal, intact, bony walls, with the buccal wall occupied by the remaining (buccal) portion of the root comprised, from inside to outside, of a thin layer of dentin, followed by cementum, periodontal ligament, and bundle bone. The implant bed was then prepared following the manufacturer's suggestions and drilling sequence, palatally to the RM, keeping the major axis of the tooth as a reference. Once again, during implant bed preparation, care was taken not to damage or mobilize the RM. As suggested in the literature [2–5], in order to obtain adequate primary stability of the implant within the postextraction socket, the preparation was extended 2-3 mm apically from the alveolus. Then, the implant (11.5 mm in length × 3.5 mm in diameter) was placed, in evident proximity to the retained buccal tooth fragment. The fixture (Anyridge®, Megagen, South Korea) inserted in the postextraction socket was conical, with knife-edge self-cutting threads, designed to improve primary stability in difficult clinical contexts [29, 30]. In addition, the fixture featured a nanostructured, calcium-incorporated surface, obtained through the incorporation of calcium ions over a classical sandblasted surface (resorbable blast media—RBM). The calcium ions were incorporated by means of a hydrothermal method, in order to accelerate the osseointegration and to improve the bone-to-implant contact (BIC%) [31, 32]. The fixture was inserted using an implant handpiece set at 20 rpm. No grafting material was placed in the space between the implant and the RM. The final abutment was placed following seating of the implant, and the fixture was immediately provisionalized with a cement-retained acrylic interim chairside restoration. Care was taken to remove all undesired centric/eccentric occlusal contacts. The patient was prescribed oral antibiotics, amoxicillin + clavulanic acid 2 g/day, for six days, and analgesic medication, ibuprofen 600 mg, for two to three days after surgery. The patient was instructed to avoid any mechanical trauma in the area for a period of two weeks and to rinse with chlorhexidine 0.2% two to three times per day, for the same period.

### 2.3. Specimen Retrieval

The fixture (#12) remained successfully in function and under loading for a period of 5 years. During all these years, the patient regularly attended the annual clinical follow-up controls and did not complain of any discomfort of complications to the implant and prosthetic restoration. A few days after reaching the fifth year from the prosthetic loading, unfortunately, the patient was the victim of a serious car accident and reported multiple fractures of the craniomaxillofacial district. In the context of a maxillofacial surgical intervention for repositioning and recomposing the fractured bones, it was necessary to remove a small maxillary bone portion that also included the area of the implant. This area appeared intact; therefore the fixture and the surrounding hard tissues were subjected to histologic examination. All procedures were carried out according to the statements/principles of the Declaration of Helsinki for medical research involving human subjects (revision of 2008).

### 2.4. Specimen Processing

The fixture and the surrounding hard tissues retrieved after a period of five years after loading were treated and processed as previously reported [31, 32], in order to facilitate histological evaluation by optical microscopy. First, the histologic specimen was washed with saline and fixed with 0.1% glutaraldehyde/4% paraformaldehyde in a 0.15 mol/L cacodylate buffer; during this process, the pH was kept at 7.4 and the temperature at 4°C. Next, a cutting machine (PreciseAutomatedOne®, Assing Technologies, Rome, Italy) cut thin sections that were dehydrated in a series of ascending alcohol rinses, inserted in resin glycolmethacrylate (Technovit7200VLC®, Heraeus, Wehrheim, Germany) and polymerized. Diamond disks and grinding machines were then used to cut the specimens to 30 *μ*m. Then the sections were stained with acid fuchsin and toluidine blue and evaluated under a polarized-light microscope (LaborluxS®, Leitz, Wetzlar, Germany). The BIC%, which was defined as the amount of mineralized bone in close contact with the surface of the fixture, was measured all around the implant surface with histomorphometry, as previously reported [31, 32]. In brief, the microscope was connected to a high-resolution camera (JVC3CCDJVCKYF55B®, JVC, Yokohama, Japan) interfaced to a powerful computer (PentiumIII1200MMX®, Intel, Santa Clara, CA, USA). A digitizing pad (D-Pad®, MatrixVision, Oppenweiller, Germany) was also employed in association with the aforementioned optical system, along with a histometry software package, capable of capturing images (ImageProPlus4.5®, Immagini&C, Milan, Italy).

## 3. Results

The retrieved tissue sample, which included the implant, the root membrane, the space between them, and the buccal bone plate, appeared intact. Only palatally to the fixture, and in the most coronal area, did it appear evident that the trauma had detached the surface of the implant from the palatal bone; that area was of less importance for the present histologic evaluation and, therefore, the sample could be considered in perfect condition for histologic and histomorphometric analysis.

**Figure 1:**
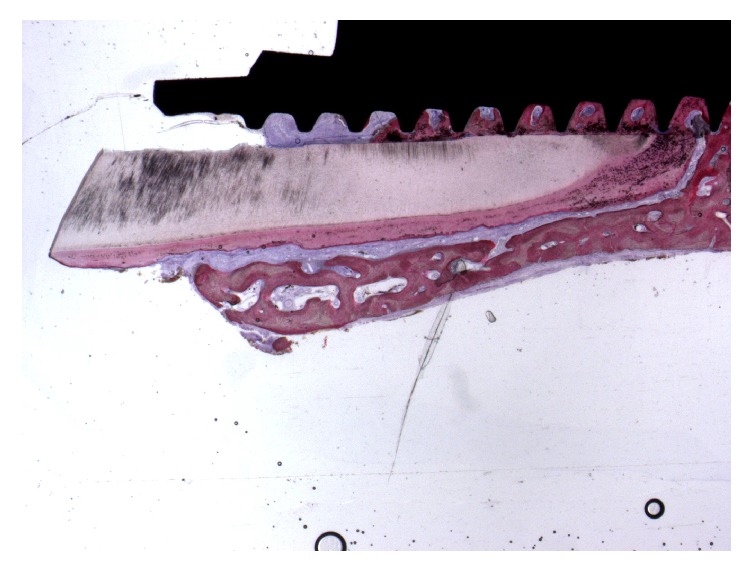
Trabecular, mature bone at the interface of the implant was observed. The bone was present between the implant and the root. The root membrane and the buccal bone plate appeared intact without any signs of resorption. Acid fuchsin-toluidine blue 12x.

The histologic analysis of the human sample revealed that, after 5 years from the placement of an immediate implant, buccal bone plate was perfectly maintained without any evidence of resorption; the buccal bone plate was supported and nourished by a healthy, intact periodontal ligament. At low magnification, the implant showed osseointegration, with a high amount of compact, mature bone on its surface (Figure 1).

**Figure 2:**
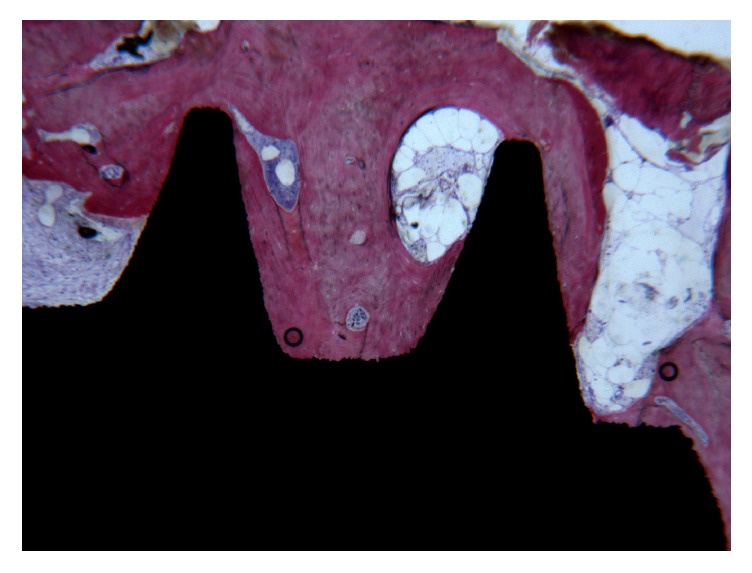
Compact bone in the medial thirds and apical portion of the implant were evident. No gaps were present at the interface. Acid fuchsin-toluidine blue 40x.

**Figure 3:**
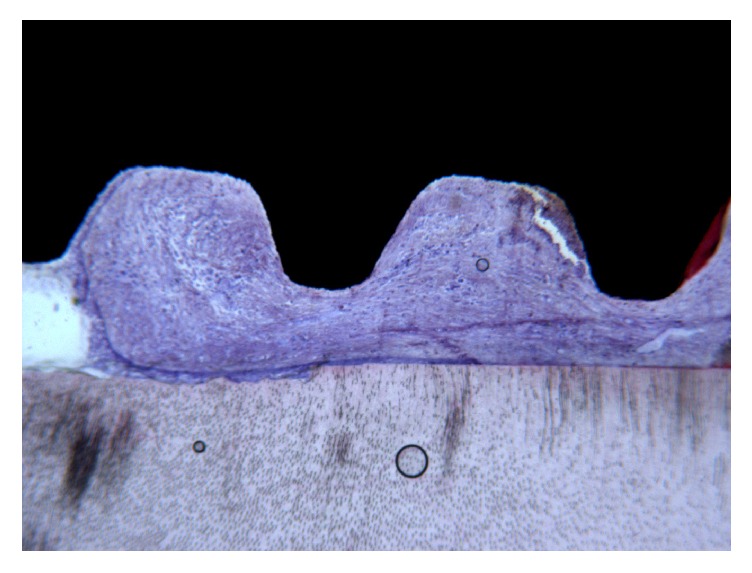
In the coronal portion, between the root and the implant, connective tissue without inflammatory infiltrate was present. Acid fuchsin-toluidine blue 40x.

With regard to the space between the RM and the implant, the apical and medial thirds were filled with compact, mature bone (Figure 2); the coronal third was colonized by noninfiltrated connective tissue (Figure 3).

**Figure 4:**
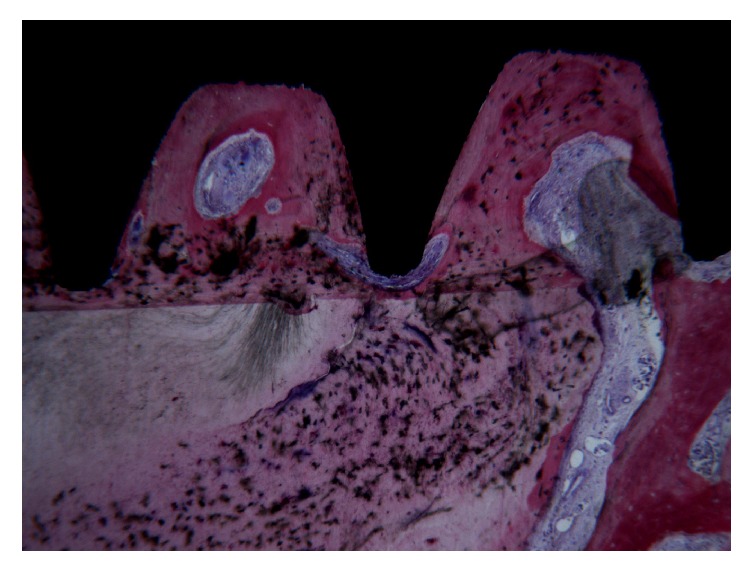
In the apical portion of the root, it was observed that the cementum migrated from the residual root to the implant surface. Acid fuchsin-toluidine blue 40x.

The root had no signs of resorption, although in the apical portion it was in direct contact with the implant surface. Interestingly, in this area, it was possible to note cementum migrated from the residual root to the implant surface (Figure 4).

The histomorphometrical evaluation showed a bone-to-implant contact of 76.2%.

## 4. Discussion

The new technique known as “socket shield” or “root membrane” (RM) is becoming more popular and is thus increasingly used by clinicians around the world as a strategy to preserve the buccal bone plate after placement of postextractive implants in the aesthetic area of the anterior maxilla [22]. Clinically, this technique appears to guarantee good results with high implant survival rates and a low incidence of complications [20, 21, 23–27]. However, it should be noted that, in the present literature, only two clinical studies have been published which include a sufficient number of patients and a sufficiently long follow-up [23, 26].

Although the clinical results obtained through the RM technique can be considered promising [23, 26], it is important to note that only a careful histological evaluation can confirm the validity of this technique, that is, the ability of the socket shield to protect the delicate buccal bone plate from resorption, in the medium and long term [22].

Unfortunately, to date only a few histological studies on the RM technique are available in the literature [19, 20, 28]. All of these are animal studies, and only one is based on a sufficient number of samples [28], since the others are reports of single cases [19, 20]. In addition, there are no histologic studies available with a longer follow-up period, as all the researches present in the literature are based on specimens retrieved only three to four months after implant placement [22].

In the first histologic report, Hürzeler et al. [19] demonstrated that retaining the buccal portion of the root during implant placement can preserve the buccal bone plate from resorption, without interfering with osseointegration. The authors proceeded as follows: first, they hemisected the third and fourth mandibular premolars (P3, P4) of a Beagle dog, keeping only the buccal fragment of the distal roots in situ, 1 mm coronal to the bone level [19]. Then, they inserted four implants, with each fixture placed lingually to the root fragment, with or without contact with it [19]. The gaps between the implants and the fragments were filled with an enamel matrix derivate, and healing abutments were attached [19]. Four months later, the dog was euthanized and histological specimens were retrieved for analysis. The histologic evaluation revealed that all fixtures were osseointegrated, without any detectable inflammatory reaction or resorption/mobility of the root fragments [19]. On the buccal side, the root fragment was attached to the buccal bone plate by means of a healthy periodontal ligament; on the lingual side, newly formed cementum was evidenced [19]. Finally, in the areas of contact between the fixtures and the fragments, new cementum was detected [19].

Bäumer et al. [20] performed a histologic study on three Beagle dogs. They hemisected the clinical crowns of the third and fourth maxillary premolars (P3, P4) and they removed the entire crowns as well as the distal root [20]. Then, they prepared the implant bed into the distal root so that a buccal segment of healthy tooth structure could remain in situ [20]. This segment was separated in a vertical direction into two pieces and the implants were inserted lingually [20]. Four months later, the animals were euthanized and the histologic samples were retrieved for analysis [20]. The authors found that the socket shield technique did not interfere with implant osseointegration, with bone formation between the implant surface and the shield; a healthy periodontal ligament was preserved on the buccal side, with no osteoclastic remodelling of the coronal part of the buccal bone plate [20]. Accordingly, the authors concluded that this method may be a valuable tool for preservation of the buccal bone plate from resorption [20].

Guirado et al. [28] performed a histologic animal study in which 36 implants were inserted in the mandible of six American Foxhound dogs, following the principles of the “root-t-belt” technique [28]. The root-t-belt technique is a modification of the technique proposed by Cherel and Etienne [33], in which the sectioning of the root is vestibular-lingual, preserving the proximal remainder of the root to protect the papilla. With the root-t-belt technique, the fixture is surrounded by root remnants, creating a belt-like structure that prevents displacement, and preserves the peri-implant bone over time [34]. In brief, the clinical crowns of the dog's third and fourth premolars (P3, P4) and the first molars (M1) of the dog's mandibles were beheaded and the roots were worn down and located at the bone crest level [28]. Implant beds were prepared in the centre of the roots, passing by 3 mm apically, and forming six groups in accordance with the remaining root thickness and remaining bone [28]. The implants were placed and, after three months, the dogs were euthanized and histologic/histomorphometric analysis was performed to investigate the stability of the crestal bone, as well as the buccal/lingual bone thickness at the implant shoulder [28]. At the end of the study, all fixtures were osseointegrated but three samples showed an inflammatory reaction, and some radicular fragments presented a small resorption process [28]. However, the radicular fragments were firmly attached on the buccal and lingual sites by means of a physiological periodontal ligament that was maintained; the preservation of the periodontal ligament contributed to stable peri-implant bone levels, with no bone resorption [28]. Finally, where spaces were present between the fixtures and the remaining root fragments, new bone formation was evidenced [28]. The authors concluded that this surgical variant of the socket shield technique may help in preserving bone and soft tissue stability, with the potential to provide aesthetic benefits when applied to patients [28].

Although all of these studies have somehow proved the validity of the technique and the ability of the root membrane to preserve the bone plates, avoiding the triggering of the bone resorption processes [19, 20, 28], it should be stressed that the evidence emerging from these researches should be considered weak, for several reasons.

First, such studies were conducted on animals, and it is not possible to directly transfer the conclusions of animal studies to the clinical context (with humans). Second, the set-up and design of these studies were different from each other, since they introduced surgical variants to the technique originally described by Hürzeler et al. [19] and revisited by Siormpas and Mitsias [26, 27]. Third, only a few histological samples were analyzed and after a short period of time (three to four months) from implant placement. This period is certainly useful for studying the early osseointegration phenomena but it does not help to clarify what may happen to the buccal bone plate in the long term, nor does it help to understand what phenomena can occur at the interface between the root membrane and the implant surface over time. Finally, for a careful evaluation of the phenomena which occur within the tissues over time, it is crucial that a human histologic sample be taken in a case where the RM procedure was intentionally performed. There is, in fact, evidence in the past literature of the integration of dental implants in direct contact with root portions casually left in the bone [35–37]; however, the data emerging from these studies should be interpreted with caution, precisely because the procedure took place unintentionally.

In our present study, the histologic analysis of a human sample retrieved after 5 years from placement of an immediate implant with the RM technique revealed that buccal bone plate was perfectly maintained without any evidence of resorption. The buccal bone plate was supported and nourished by a healthy, intact periodontal ligament. The implant showed osseointegration, with a high amount of compact, mature bone on its surface; the histomorphometric analysis found a bone-to-implant contact percentage (BIC%) of 76.2%. Moreover, most of the space between the implant and the membrane was filled with compact, mature bone, and only the coronal part (the most coronal implant threads) showed the presence of a noninfiltrated connective tissue. Finally, the root itself appeared intact with no signs of resorption, although in the apical portion it was in direct contact with the implant surface. In the most apical portion of the sample, it was possible to note cementum migrated from the residual root to the implant surface.

Our study has clear advantages over other histologic studies published so far in the literature. First, it is a human study: the evidence that emerges from human histologic studies has higher value than that emerging from animal histologies [31, 32, 38]. Obviously, it is very difficult to obtain such human samples for ethical reasons: in our case, this was possible due to a severe trauma which occurred to the patient, with multiple fractures, which allowed the removal, during maxillofacial surgery for fracture recomposition and fixation, of a small bone portion. Second, our specimen was retrieved after five years of function; therefore it allowed us to gather data on hard tissue stability, as well as on the interface between the RM and the implant, in the medium term. Third, our histological study examines the results obtained in a case where the RM technique was intentionally performed. Our data, in fact, refer to a postextraction implant performed in the aesthetic area of the maxilla, according to the conventional RM technique, which was intentionally performed. It is not an occasional finding, obtained by analyzing a histologic sample in which a fixture was unintentionally placed close to a root fragment [35–37]. In this specific case, the vestibular shield was left 1 mm above the bone, as originally reported in the classical socket shield technique. The reason for not reducing the root to the level of, or even below, the bone crest was to maintain the dentogingival fibres intact for enhancing soft tissue aesthetics. In addition, as preferred by the authors [26, 27], no grafting material was used to fill the gap between the shield and the implant. The presence of mature connective tissue in the most coronal part of the space between the implant and the membrane could suggest the use of grafting material to prevent this area being colonized by soft tissues in the immediate postsurgical period. In fact, at this stage, there is a competition between different tissues to colonize this space. The use of grafting material could help, but at the same time it may pose a risk of infection and cause a slowdown in the healing processes. It is important to note that in our specimen we found a large amount of mature, compact bone in the apical and mean portion of the gap between the membrane and the implant surface; if grafting material is used, it is difficult to obtain a bone of such quality. In addition, the connective tissue found in our specimen is noninfiltrated, and the absence of inflammation in this area can be considered a positive aspect.

Our present study has limitations: in fact, it is a report from one single case, and the analysis of several histologic specimens would be preferable to draw more specific conclusions about the effectiveness of the RM technique in preserving the buccal bone plate over time. In particular, a randomized controlled human histologic study would be needed to positively confirm the validity of this surgical technique, and to understand whether the use of grafting material in the space between the membrane and the implant is advisable. Therefore, further histologic and histomorphometric studies are needed to investigate the tissues dynamics at the bone-implant interface.

## 5. Conclusions

Our present human histologic study supports the assertion that the RM technique is effective in preventing bone resorption of the buccal bone plate of the anterior maxilla, five years after the placement of an immediate implant. This human histologic evidence that RM can preserve the buccal bone plate is of great value since it can help validate the clinical use of this surgical technique to maintain the hard and soft tissues over time and to optimize aesthetic results. Further studies will be needed to confirm such evidence and to understand whether the placement of grafting material in the space between the membrane and the implant is actually advisable.
